# One-Step Generation of Multiple Gene-Edited Pigs by Electroporation of the CRISPR/Cas9 System into Zygotes to Reduce Xenoantigen Biosynthesis

**DOI:** 10.3390/ijms22052249

**Published:** 2021-02-24

**Authors:** Fuminori Tanihara, Maki Hirata, Nhien Thi Nguyen, Osamu Sawamoto, Takeshi Kikuchi, Takeshige Otoi

**Affiliations:** 1 Faculty of Bioscience and Bioindustry, Tokushima University, 2272-1 Ishii, Myozai-gun, Tokushima 779-3233, Japan; f_tanihara@jichi.ac.jp (F.T.); mhirata@tokushima-u.ac.jp (M.H.); nhienvet@gmail.com (N.T.N.); 2 Research and Development Center, Otsuka Pharmaceutical Factory, Inc., 115 Muya-cho, Naruto, Tokushima 772-8601, Japan; Sawamoto.Osamu@otsuka.jp (O.S.); Kikuchi.Takeshi@otsuka.jp (T.K.)

**Keywords:** CRISPR/Cas9, *GGTA1*, *CMAH*, *B4GALNT2*, pig, in vitro fertilization, electroporation

## Abstract

Xenoantigens cause hyperacute rejection and limit the success of interspecific xenografts. Therefore, genes involved in xenoantigen biosynthesis, such as *GGTA1*, *CMAH*, and *B4GALNT2*, are key targets to improve the outcomes of xenotransplantation. In this study, we introduced a CRISPR/Cas9 system simultaneously targeting *GGTA1*, *CMAH,* and *B4GALNT2* into in vitro-fertilized zygotes using electroporation for the one-step generation of multiple gene-edited pigs without xenoantigens. First, we optimized the combination of guide RNAs (gRNAs) targeting *GGTA1* and *CMAH* with respect to gene editing efficiency in zygotes, and transferred electroporated embryos with the optimized gRNAs and Cas9 into recipient gilts. Next, we optimized the Cas9 protein concentration with respect to the gene editing efficiency when *GGTA1*, *CMAH*, and *B4GALNT2* were targeted simultaneously, and generated gene-edited pigs using the optimized conditions. We achieved the one-step generation of *GGTA1*/*CMAH* double-edited pigs and *GGTA1*/*CMAH/B4GALNT2* triple-edited pigs. Immunohistological analyses demonstrated the downregulation of xenoantigens; however, these multiple gene-edited pigs were genetic mosaics that failed to knock out some xenoantigens. Although mosaicism should be resolved, the electroporation technique could become a primary method for the one-step generation of multiple gene modifications in pigs aimed at improving pig-to-human xenotransplantation.

## 1. Introduction

Demand for organ transplantation has increased substantially as a result of the contemporary prolongation of life expectancy and corresponding increases in the incidence of chronic diseases and end-stage organ failure [1,2]. Xenotransplantation is one solution to overcome the shortage of organs for human transplantation. Pig organs are ideal for this purpose owing to their close similarity to human organs, especially in terms of size and structure. However, multiple hurdles, including immunological barriers, need to be overcome for their utilization as an alternative tissue source. In particular, xenoantigens limit the success of interspecific xenografts. Antibody–xenoantigen complexes lead to complement activation and immediate hyperacute rejection [3]. The galactosyl-alpha 1,3-galactose (Galα(1,3)Gal) epitope [4], glycans modified with *N*-glycolylneuraminic acid (Neu5Gc) [5], and Sd^a^ [6] are the major carbohydrate xenoantigens expressed in porcine tissues causing hyperacute rejection. Galα(1,3)Gal is not expressed in humans but is expressed on the surface of porcine endothelial cells [2,4]. The biosynthesis of Galα(1,3)Gal is regulated by α1,3-galactosyltransferase, encoded by glycoprotein galactosyltransferase alpha 1,3 (*GGTA1*) [4]. Inactivation of Galα(1,3)Gal is the first step in successful xenotransplantation. Furthermore, Neu5Gc synthesized by cytidine monophospho-*N*-acetylneuraminic acid hydroxylase (CMAH) [5,7] and Sd^a^ synthesized by beta-1,4-*N*-acetyl-galactosaminyltransferase 2 (B4GALNT2) [6] need to be eliminated to reduce pig organ rejection and prolong survival. Humans, but not non-human primates, make an array of antibodies to Neu5Gc-modified glycans [8,9]. Sd^a^ has been examined as a non-Gal porcine xenogeneic antigen and B4GALNT2 is expressed in major vascularized organs [6]. Humans typically produce low levels of antibodies against Sd^a^ [10].

Genetically modified pigs without these xenoantigens will dramatically improve the success of pig-to-human xenotransplantation. *GGTA1*, *CMAH,* and *B4GALNT2* triple-knockout pigs generated using somatic cell nuclear transfer (SCNT) significantly reduced human IgG and IgM antibody binding to porcine peripheral blood monocytes, red blood cells [11,12,13], and the pericardium [14]. However, SCNT requires sophisticated techniques, including micromanipulator systems for the nuclear transfer of donor cells [15]. In mice, electroporation is widely used to introduce the CRISPR/Cas9 system, consisting of Cas9 and a guide RNA (gRNA), into zygotes, resulting in efficient gene editing [16]. We established the GEEP (“gene editing by electroporation of Cas9 protein”) method [17] for electroporation-mediated gene editing using the CRISPR/Cas9 system in porcine zygotes without complicated micromanipulation techniques. We previously generated pig models of disease [18,19], as well as *GGTA1*-deficient pigs using GEEP [20]. However, as described above, all major xenoantigens expressed in porcine tissues should be removed for successful xenotransplantation.

Establishing multiple gene-knockout pig lines by mating pigs carrying a mutation in a single gene is time-consuming and costly. Therefore, the one-step generation of multiple gene-disrupted pigs will dramatically accelerate subsequent phenotypic analyses and provide a more realistic approach for improving outcomes in xenotransplantation. Although we have previously demonstrated multiple gene editing using GEEP [21,22], the generation of multiple gene-edited offspring from electroporated zygotes has not yet been attempted. In this study, we generated multiple gene-edited pigs from zygotes electroporated with gRNAs targeting two genes (*GGTA1* and *CMAH*) and three genes (*GGTA1*, *CMAH*, and *B4GALNT*). Our results support the use of GEEP for the one-step establishment of genetically modified pigs, which may serve as a resource for pig-to-human xenotransplantation studies.

## 2. Results

### 2.1. Generation of GGTA1/CMAH Double-Edited Pigs

**Figure 1:**
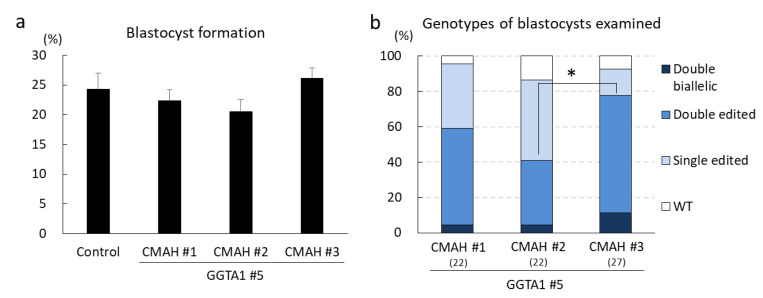
Optimization of gRNA combinations targeting *GGTA1* and *CMAH*. (**a**) Blastocyst formation rates of electroporated zygotes. For each treatment group, five replicates with 263–268 oocytes per treatment were analyzed. Means ± SEM are shown. (**b**) Genotypes of blastocysts were determined using TIDE. Numbers within parentheses indicate the total numbers of examined blastocysts. Percentages of blastocysts carrying mutations in *GGTA1* and *CMAH* were analyzed using chi-squared tests. * *p* < 0.05. WT, wild-type; Single edited, blastocysts carrying a mutation in *GGTA1* or *CMAH*; Double-edited, blastocysts carrying mutations in *GGTA1* and *CMAH*; Double biallelic, blastocysts carrying biallelic mutations in *GGTA1* and *CMAH*.

We previously optimized an efficient gRNA targeting *GGTA1* (GGTA1#5) (Table S1) and successfully generated *GGTA1* biallelic mutant pigs using this gRNA [20]. For *CMAH* gene editing, two efficient gRNAs (CMAH#1 and #2) have been confirmed using in vitro-fertilized zygotes [21]. We designed an additional gRNA targeting *CMAH* (CMAH#3) (Table S1) and evaluated the effects of combinations of gRNAs on embryonic development and gene editing efficiency. GGTA1#5 and CMAH#1, #2, or #3 were each added at a concentration of 100 ng/μL and introduced into in vitro-fertilized zygotes using electroporation (five 1 ms square pulses at 25 V), along with 100 ng/μL Cas9. We analyzed the blastocyst formation rate from electroporated embryos. Additionally, the genotypes of blastocysts were determined using Sanger sequencing. The TIDE (tracking of indels by decomposition) bioinformatics package [23] was used to determine the genome editing efficiency of each gRNA combination (Figure 1). In this study, blastocysts carrying more than one type of mutation and the wild-type (WT) sequence were defined as mosaics. The blastocyst formation rate did not differ significantly among zygotes treated with different gRNA combinations (Figure 1a). However, the rate of blastocysts carrying mutations in both *GGTA1* and *CMAH* was significantly higher (*p* < 0.05) for zygotes with GGTA1#5 and CMAH#3 than for zygotes with GGTA1#5 and CMAH#2 (Figure 1b). When we evaluated mutations introduced into each targeting gene, the rate of blastocysts carrying biallelic mutations in the *CMAH* gene was also higher for zygotes with CMAH#3 than for zygotes with CMAH#1 or CMAH#2 (Figure S1). Therefore, we used GGTA1#5 and CMAH#3 to generate *GGTA1*/*CMAH* double-edited pigs.

**Figure 2:**
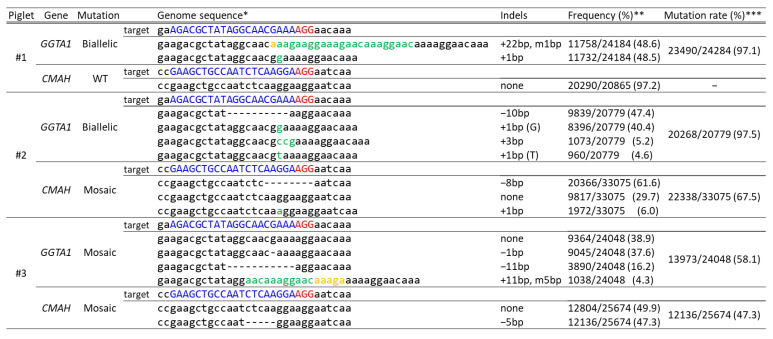
Deep sequencing analysis of the *GGTA1* and *CMAH* target regions in delivered piglets. * Blue and red indicate the target sequences and protospacer adjacent motif (PAM) sequences of each gRNA, respectively. Green and yellow represent inserted and modified sequences, respectively. ** The frequency was defined as the ratio of the number of amplicons to the total read number. *** The mutation rate was defined as the ratio of the total number of mutant amplicons to the total read number. WT, wild-type.

**Figure 3:**
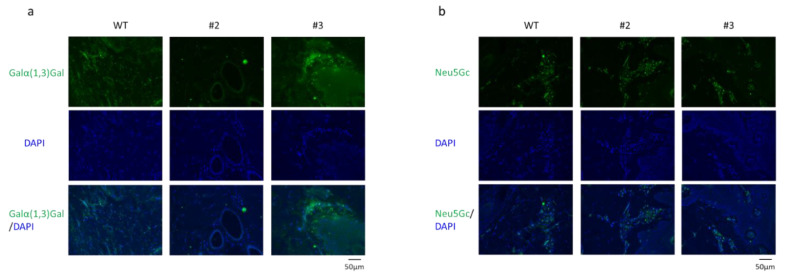
Immunohistochemical assessment of wild-type and *GGTA1*/*CMAH* double-edited piglets. Ear biopsies derived from wild-type (WT) and *GGTA1*/*CMAH* double-edited piglets (#2 and #3) were immunohistochemically stained for Galα(1,3)Gal (green) (**a**) and Neu5Gc (green) (**b**). These tissues were counterstained with 4’, 6-diamidino-2-phenylindole (DAPI) (blue). The scale bar in each panel represents 50 μm.

Cas9 and two gRNAs (GGTA1#5 and CMAH#3) were introduced into zygotes using electroporation. These zygotes were then transferred into the oviducts of three recipient gilts (approximately 200 zygotes/gilts). One recipient gilt became pregnant and gave birth to three piglets. A deep sequencing analysis of DNA samples derived from ear biopsy samples of the delivered piglets was performed to evaluate the gene editing efficiency. Two of the three piglets (#2 and #3) carried mutations in both *GGTA1* and *CMAH* (Figure 2). Piglets #1 and #2 had no WT sequences in the *GGTA1* genomic regions flanking the target sites; therefore, they were considered *GGTA1* biallelic mutants. However, we could not obtain *CMAH* biallelic mutant pigs. The expression levels of the Galα(1,3)Gal and Neu5Gc epitopes were assessed by staining with Alexa 488-labeled isolectin B4 and an anti-Neu5Gc antibody, respectively, in ear biopsy tissues derived from *GGTA1* and *CMAH* double-edited pigs (#2 and #3). The histological analysis indicated a GGTA1 deficiency in piglet #2 (Figure 3a). The expression of Galα(1,3)Gal in piglet #3 carrying a mosaic mutation in *GGTA1* was similar to that in the WT. The expression of the Neu5Gc epitope in piglets #2 and #3 carrying a mosaic mutation in *CMAH* was also similar to that in the WT (Figure 3b).

### 2.2. Generation of GGTA1/CMAH/B4GALNT Triple-Edited Pigs

**Figure 4:**
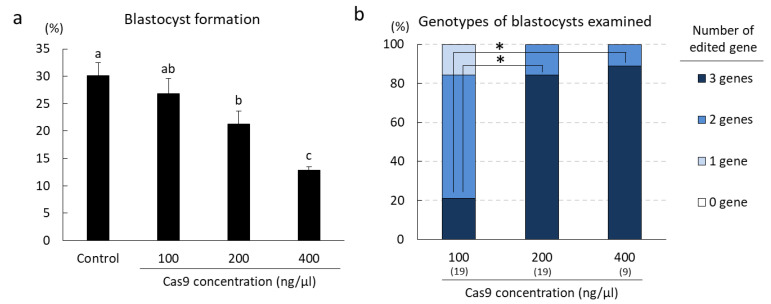
Optimization of conditions for gene editing targeting *GGTA1*, *CMAH,* and *B4GALNT2*. (**a**) Effects of the Cas9 concentration on the developmental competence of electroporated zygotes. For each treatment group, five replicates with 243–253 oocytes per treatment were analyzed. Means ± SEM are shown. ^a–c^ Values with different superscript letters differed significantly (*p* < 0.05) and those sharing the same letter did not differ significantly. (**b**) Effects of the Cas9 concentration on gene editing efficiency. Genotypes of blastocysts were determined using TIDE. Numbers within parentheses indicate the total numbers of examined blastocysts. Percentages of blastocysts carrying mutations in *GGTA1*, *CMAH*, and *B4GALNT2* were analyzed using chi-squared tests. * *p* < 0.05.

We previously validated a highly efficient gRNA targeting *B4GALNT2* [24], referred to as B4GALNT2#1 (Table S1). To improve the gene editing efficiency by targeting three genes, we evaluated the effects of the Cas9 concentration on gene editing via the GEEP method. GGTA1#5, CMAH#3, and B4GALNT2#1 were mixed at a concentration of 100 ng/μL and introduced into the in vitro-fertilized zygotes using electroporation along with 100 ng/μL, 200 ng/µL, or 400 ng/µL of Cas9. The gene editing efficiency was affected by the Cas9 concentration. The rates of blastocysts carrying mutations in all targeted genes were significantly higher (*p* < 0.05) in embryos electroporated with 200 and 400 ng/µL Cas9 than in embryos electroporated with 100 ng/µL Cas9 (Figure 4b). When we evaluated mutations introduced into each targeting gene, the rate of blastocysts carrying biallelic mutations and the gene editing efficiency in the mutant blastocysts also increased as the Cas9 concentration increased in *CMAH* and *B4GALNT2* genes (Figure S2). However, the blastocyst formation rate of embryos electroporated with 400 ng/µL Cas9 was significantly lower (*p* < 0.05) than that of embryos electroporated with 100 ng/µL Cas9 (Figure 4a), indicating that the electroporation of 400 ng/µL Cas9 was harmful to subsequent embryonic development. Therefore, a Cas9 concentration of 200 ng/µL was optimal to generate *GGTA1*/*CMAH*/*B4GALNT2* triple-edited pigs in this study.

**Figure 5:**
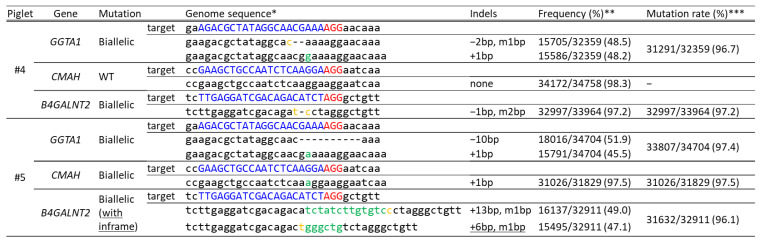
Deep sequencing analysis of the *GGTA1*, *CMAH*, and *B4GALNT2* target regions in delivered piglets. * Blue and red indicate the target sequences and PAM sequences of each gRNA, respectively. Green and yellow indicate inserted and modified sequences, respectively. ** The frequency was defined as the ratio of the number of amplicons to the total read number. *** The mutation rate was defined as the ratio of the total number of mutant amplicons to the total read number. WT, wild-type. Underlining indicates the presence of an inframe mutation.

Approximately 200 zygotes were electroporated in a solution containing 200 ng/µL Cas9, 100 ng/µL GGTA1#5, 100 ng/µL CMAH#3, and 100 ng/µL B4GALNT2#1. These zygotes were then transferred into the oviducts of one recipient gilt. The recipient gilt became pregnant and gave birth to two piglets (piglets #4 and #5). A deep sequencing analysis of DNA samples derived from ear biopsy samples of the delivered piglets revealed that piglet #4 carried biallelic mutations in *GGTA1* and *B4GALNT2*, and piglet #5 carried mutations in all three of the target genes (Figure 5). However, the mutation in *B4GALNT2* in piglet #5 included an inframe.

**Figure 6:**
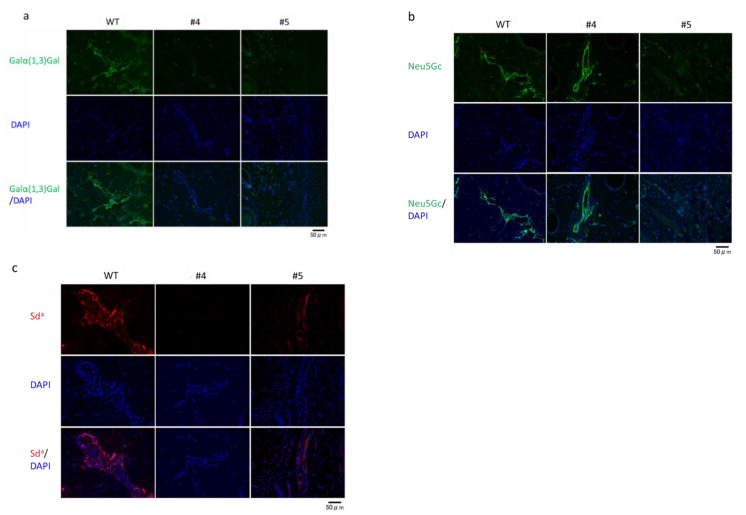
Immunohistochemical assessment of wild-type, *GGTA1*/*B4GALNT2* double-edited (#4), and *GGTA1*/*CMAH/B4GALNT2* triple-edited (#5) piglets. The ear biopsies derived from wild-type (WT) and mutant piglets (#4 and #5) were immunohistochemically stained for Galα(1,3)Gal (green) (**a**), Neu5Gc (green) (**b**), and Sd^a^ (red) (**c**). These tissues were counterstained with 4′, 6-diamidino-2-phenylindole (DAPI) (blue). The scale bar in each panel represents 50 μm.

Next, we evaluated the expression levels of Galα(1,3)Gal, Neu5Gc, and Sd^a^ epitopes (Figure 6). In piglet #4, levels of Galα(1,3)Gal and Sd^a^ epitopes were lower than those in WT controls, and the expression of the Neu5Gc epitope was observed. In piglet #5, the downregulation of Galα(1,3)Gal and Neu5Gc epitopes indicated a deficiency in GGTA1 and CMAH (Figure 6a,b). The expression of the Sd^a^ epitope in piglet #5 carrying an inframe mutation was similar to that in the WT (Figure 6c).

## 3. Discussion

In this study, based on the CRISPR/Cas9 system, we successfully generated *GGTA1*/*CMAH* double-edited and *GGTA1*/*CMAH*/*B4GALNT2* triple-edited pigs using the GEEP method. Multiple gene-edited animals have been generated using the cytoplasmic microinjection of CRISPR/Cas9 in mice [25,26], rats [27], and monkeys [28]. In pigs, the SCNT technique is a primary method for generating multiple gene-modified pigs, while one-step multiple gene editing during embryogenesis has been achieved by microinjection alone [29]. To the best of our knowledge, this is the first demonstration of the generation of multiple gene-edited pigs from CRISPR/Cas9-mediated gene-edited zygotes using electroporation, which is a simple and micromanipulation-free method.

First, we generated *GGTA1*/*CMAH* double-edited pigs after evaluating the effects of gRNA combinations on embryonic viability and the gene editing efficiency using in vitro cultured blastocysts. Cross-talk between gRNAs, in which a gRNA affects the gene editing efficiency of another gRNA, is a concern. In our previous study, when porcine zygotes were electroporated with pooled gRNAs targeting multiple genes, the mutation rates in each gene were lower than those after electroporation with single gRNAs [21]. In this study, we used gRNAs (GGTA1#5, CMAH#1 and #2) that induce gene editing in zygotes with high efficiency when used individually. However, this study demonstrated that the simultaneous introduction of gRNAs targeting *GGTA1* and *CMAH* reduces the rate of blastocysts carrying biallelic mutations compared with the single-use results of our previous study [20,21]. CMAH#3, which is a newly designed gRNA introduced in this study, showed a higher biallelic mutation rate compared with CMAH#1 and #2 (Figure S1). When low gene-editing results, presumably due to cross-talk between gRNAs, were observed during multiple gene targeting, a redesign of the gRNA can be one of the approaches used to improve efficiency. On the other hand, the combination of gRNAs targeting *GGTA1* and *CMAH* did not affect the developmental competence of electroporated zygotes. Therefore, we selected a gRNA combination that maximized the gene editing efficiency. After the transfer of electroporated zygotes with gRNAs, two of three piglets carried mutations in the *GGTA1* and *CMAH* genes; however, mosaicism prevented the inactivation of the Neu5Gc epitope. An immunohistochemical analysis revealed the expression of Neu5Gc in both *GGTA1*/*CMAH* double-edited pigs. Therefore, to further optimize the conditions for gene editing, we evaluated various Cas9 concentrations for the generation of *GGTA1*/*CMAH*/*B4GALNT2* triple-edited pigs using GEEP in Experiment 2.

The interference of gene editing, presumably due to cross-talk between gRNAs, was also observed in Experiment 2. The simultaneous introduction of gRNA targeting *B4GALNT2* induced a lower biallelic mutation rate compared with our previous study in which gRNAs were used individually [24]. gRNA targeting *CMAH* also demonstrated fewer gene-editing events when used to induce triple gene editing compared with when it was used to induce double editing in Experiment 1. Both were introduced into zygotes with 100 ng/µl Cas9 (Figure S2). Elevation of the Cas9 concentration improves the rate of blastocysts carrying biallelic mutations and the gene editing efficiency in the mutant blastocysts. We confirmed that electroporation with 200 ng/µL Cas9 induced mutations in three targeted genes with high efficiency and minimal effects on embryonic development. In this study, although the amount of gRNA was kept constant, gene editing efficiency was improved via the addition of more Cas9 protein. This result indicates that gRNAs are enough for our electroporation-mediated introduction. Thus, adding more Cas9 resulted in an increase in the amount of active CRISPR/Cas9 ribonucleoprotein. In our previous study, we investigated the effect of various concentrations of Cas9 (0 to 1000 ng/µL) on the development and gene editing of porcine embryos via electroporation using a single gRNA targeting the myostatin gene [30]. The biallelic mutation rate and editing efficiency increased as the Cas9 protein concentration increased. However, the development of blastocysts from electroporated zygotes was not affected by the Cas9 concentration, in contrast with the results of this study. One explanation for the decrease in blastocyst formation rates in our study is that multiple gene editing may affect embryo viability. In our previous study, targeting four genes simultaneously did not negatively affect blastocyst formation [21], presumably because the mutation rate in each gene was lower than that for electroporation with single gRNAs. In mice, quintuple gene modification has been achieved by the microinjection of the CRISPR/Cas9 system into one-cell stage embryos [26]; in this study, the number of pups decreased as the gene editing efficiency increased. On the other hand, triple-mutated rats have been generated by the co-microinjection of three gRNAs targeting different genes without affecting embryonic viability [27]. The combination of gRNAs, concentrations of gRNAs and Cas9 protein/mRNA, targeting genes, and animal species are possible factors affecting the viability of zygotes and embryos following gene editing.

After the embryo transfer of zygotes with three gRNAs along with Cas9, we generated *GGTA1*/*CMAH*/*B4GALNT2* triple-edited pigs under optimized conditions. However, the triple-edited pigs also carried a mosaic mutation. Avoiding mosaicism is crucial with respect to time, labor, and costs, especially in pigs, as large experimental animals, because mosaicism in the founder generation requires subsequent breeding for stable phenotype expression. Gene editing of zygotes/embryos using the CRISPR/Cas9 system poses a risk of mosaicism [17,31]. In one study using rats, the CRISPR/Cas9 system introduced into in vitro-fertilized embryos using electroporation disrupted genes with 100% efficiency [32]. In the case of marmoset embryos manipulated by the cytoplasmic microinjection of gene editors, optimized conditions using transcription activator-like effector nucleases (TALENs) achieved highly efficient gene disruption with low or no mosaicism [33], whereas higher mosaicism was observed when the CRISPR/Cas9 system was used [34]. Further optimization of gene editing using gene editors is required in pigs.

In this study, in order to reduce mosaicism, we optimized the Cas9 concentration. However, this approach may affect off-target events. Off-target effects, such as unexpected DNA cleavage caused by the binding of gene editors to unintended genomic sites, is a major concern in gene editing. Ryczek et al. confirmed off-target mutations induced by CRISPR/Cas9 system after targeting xenotransplantation-related genes including *GGTA1*, *CMAH*, and *B4GALNT2* [35]. In this study, we designed gRNAs using the COSMID webtool to minimize off-target effects; however, further strategies are crucial, especially for clinical applications in humans requiring precise gene modification.

To minimize the off-target effects and improve practical gene editing, the latest approaches were developed, e.g., off-target detection by algorithmically-designed software and genome-wide assays, cytosine or adenine base editors, prime editing, Cas9 variants including dCas9 and Cas9 paired nickase, and the chemical modification of gRNA [36]. A high-fidelity Cas9 mutant also resulted in high on-target activity while reducing off-target effects in human cells [37]. Furthermore, well-designed gRNA with high specificity using tools to detect and evaluate the gRNA efficiency will reduce the labor required to analyze off-target candidates [36,38]. gRNAs should be carefully designed to minimize the likelihood of off-target effects; however, the potential for off-target events cannot be completely eliminated. In our previous study, although the increasing concentration of CRISPR/Cas9 components was effective in increasing gene editing efficiency without off-target events [30], the frequency of mosaicism has the potential to vary with the organs derived from founder pigs [20]. We should minimize off-target events by utilizing the latest developed strategies in founder generations, and evaluate possible off-target events of non-mosaic genetically-modified donor lines for xenotransplantation prior to a clinical application for humans.

*GGTA1*/*CMAH*/*B4GALNT2*-deficient pigs are a promising resource for pig-to-human xenotransplantation. Furthermore, the expression of human complement regulators in pigs is one approach to control complement activation, which induces hyperacute rejection [2,39]. As a next step, multiple gene modifications, including knock-ins of human genes, will be essential to prolong the function of xenotransplanted pig organs in humans. Using electroporation with the CRISPR/Cas9 system, the knock-in of long single-stranded DNAs (600 to 1500 bases) has been successfully demonstrated in mice [40]. However, in pigs, the size of molecules introduced into zygotes or embryos is limited because porcine in vitro-fertilized zygotes have greater sensitivity to electricity than that of in vivo-derived mouse embryos [17,41]. A high electroporation voltage to introduce large molecules has harmful effects on embryonic viability [41]. To deliver the knock-in donor DNA into zygotes, an adeno-associated viral (AAV) vector has been applied in mice [42] and rats [32] without removing the zona pellucida, requiring no sophisticated techniques. In pigs, the development of new, efficient delivery techniques of large molecules for zygotes and embryos is crucial.

In conclusion, the GEEP method can be used to generate multiple gene-modified pigs. Although mosaicism should be resolved, this simple technique could become a primary method for the one-step generation of multiple gene modifications using the CRISPR/Cas9 system.

## 4. Materials and Methods

### 4.1. Animals

The animal experiments were approved by the Institutional Animal Care and Use Committee of Tokushima University (approval number: T2019-11, date of approval: 9 April 2019). All animal care and experimental procedures, including the determination of experimental endpoints, were performed in accordance with the Guidelines for Animal Experiments of Tokushima University. All animals were housed and maintained in accordance with Institutional Animal Care and Use Committee guidelines. Four sexually mature Landrace gilts were obtained from the Tokushima Prefectural Livestock Research Institute (Tokushima, Japan). Pigs were housed in a temperature-controlled room (25 ± 3 °C) under a 12 h light/12 h dark cycle with free access to water and were provided with commercial feed (JA Nishinihon Kumiai Shiryou, Hyogo, Japan). The health condition of each pig was observed daily by the animal husbandry staff under the supervision of an attending veterinarian. To minimize animal suffering, all surgical procedures were performed under anesthesia by intramuscular injection of 10 mg/kg ketamine (Ketalar, ketamine hydrochloride, Daiichi Sankyo Pharmaceutical, Tokyo, Japan) and continuous inhalation of 2% to 3% isoflurane (Mylan, Osaka, Japan) in the operating room. Euthanasia was performed by intravenous injection of a potassium chloride solution (3 mmol/kg) under deep anesthesia by isoflurane according to the American Veterinary Medical Association Guidelines for the Euthanasia of Animals.

### 4.2. Oocyte Collection, In Vitro Maturation, and Fertilization

Pig ovaries were obtained from prepubertal gilts (Landrace × Large White × Duroc breeds) at a local slaughterhouse and were transported in physiological saline within 1 h to the laboratory at 30 °C. Ovaries were washed three times with prewarmed physiological saline solution supplemented with 100 IU/mL penicillin G potassium (Meiji, Tokyo, Japan) and 0.1 mg/mL streptomycin sulfate (Meiji). Follicles with diameters of 3–6 mm on the ovarian surface were placed on a sterilized dish using a surgical blade, and cumulus–oocyte complexes (COCs) were visualized and collected under a stereomicroscope. Approximately 50 COCs were cultured in 500 µL of maturation medium consisting of tissue culture medium 199 with Earle’s salts (TCM 199; Gibco/Invitrogen Co., Carlsbad, CA, USA) supplemented with 10% (*v*/*v*) porcine follicular fluid, 0.6 mM cysteine (Sigma-Aldrich, St. Louis, MO, USA), 50 µM β-mercaptoethanol (Wako Pure Chemical Industries Ltd., Osaka, Japan), 50 µM sodium pyruvate (Sigma-Aldrich), 2 mg/mL d-sorbitol (Wako Pure Chemical Industries Ltd.), 10 IU/mL equine chorionic gonadotropin (eCG; Kyoritu Seiyaku, Tokyo, Japan), 10 IU/mL human chorionic gonadotropin (hCG; Kyoritu Seiyaku), and 50 µg/mL gentamicin (Sigma-Aldrich), then covered with mineral oil (Sigma-Aldrich) for 22 h in 4-well dishes (Nunc A/S, Roskilde, Denmark). The COCs were transferred into maturation medium without hormones for an additional 22 h. COCs were incubated at 39 °C in a humidified incubator containing 5% CO_2_.

The matured oocytes were subjected to in vitro fertilization as described previously [43]. Briefly, frozen–thawed ejaculated spermatozoa collected from WT boar were transferred into 5 mL of porcine fertilization medium (PFM; Research Institute for the Functional Peptides Co., Yamagata, Japan) and washed using centrifugation at 500× *g* for 5 min. The pelleted spermatozoa were resuspended in fertilization medium and adjusted to a density of 1 × 10^6^ cells/mL. Approximately 50 oocytes were transferred to 500 µL of sperm-containing fertilization medium, covered with mineral oil in 4-well dishes, and co-incubated for 5 h at 39 °C in a humidified incubator containing 5% CO_2,_ 5% O_2_, and 90% N_2_. After co-incubation, the putative zygotes were denuded from the cumulus cells and attached spermatozoa by mechanical pipetting, transferred to porcine zygote medium (PZM-5; Research Institute for the Functional Peptides Co.), and cultured for 7 h until electroporation.

### 4.3. Electroporation

Electroporation was performed as described previously [17]. Briefly, an electrode (LF501PT1-20; BEX, Tokyo, Japan) was connected to a CUY21EDIT II electroporator (BEX) and was set under a stereoscopic microscope. The 50 inseminated zygotes were washed with Opti-MEM I solution (Gibco/Invitrogen, Carlsbad, CA, USA) and placed in a line in the electrode gap in a chamber slide filled with 10 μL of nuclease-free duplex buffer (IDT; Integrated DNA Technologies, Coralville, IA, USA) containing gRNA (Alt-R CRISPR crRNAs and tracrRNA, chemically modified and length-optimized variants of the native guide RNAs purchased from IDT), and Cas9 protein (Guide-it Recombinant Cas9; Takara Bio, Shiga, Japan). According to the manufacturer’s instructions, crRNA contains chemical modifications to protect it from degradation by cellular RNases, and tracrRNA contains chemical modifications conferring high nuclease resistance. gRNAs were designed using the CRISPRdirect webtool (https://crispr.dbcls.jp/ accesed date: 25 January 2021) [44]. To minimize off-target effects, the 12 bases at the 3’ end of the designed gRNAs had no identical sequence matches to the pig genome other than the target regions of *GGTA1*, *CMAH*, and *B4GALNT2,* as determined using the COSMID webtool (https://crispr.bme.gatech.edu/ accesed date: 25 January 2021), which scores and ranks off-target candidate sequences based on locations and numbers of base mismatches, deletions, and insertions, when compared to the gRNA sequence [45].

After electroporation (five 1 ms square pulses at 25 V), the zygotes were washed with PZM-5 and cultured until embryo transfer (for 12 h) or for 3 days. The embryos that were cultured for 3 days were subsequently incubated in porcine blastocyst medium (PBM; Research Institute for the Functional Peptides Co.) for 4 days to evaluate their ability to develop to the blastocyst stage and for blastocyst genotyping. As a control, some of the inseminated zygotes were cultured with PZM-5 and PBM for 7 days without electroporation. Zygotes and embryos were incubated at 39 °C in a humidified incubator containing 5% CO_2_, 5% O_2_, and 90% N_2_.

### 4.4. Analysis of the Targeted Gene after Electroporation

Genomic DNA was isolated from blastocysts by boiling in a 50 mM NaOH solution. After neutralization, the genomic regions flanking the gRNA target sequences were PCR-amplified using specific primers (Table S1). PCR products were extracted by agarose gel electrophoresis. The targeted genomic regions were directly sequenced using Sanger sequencing with the BigDye Terminator Cycle Sequencing Kit ver. 3.1 (Thermo Fisher Scientific, Waltham, MA, USA) and the ABI 3500 Genetic Analyzer (Applied Biosystems, Foster City, CA, USA). The TIDE bioinformatics package [23] was used to determine the genotypes of blastocysts, which were classified as having biallelic mutations (carrying no WT sequences), mosaics (carrying more than one type of mutation and the WT sequence), or WT (carrying only the WT sequence). Gene editing efficiency was defined as the proportion of indel mutation events in the blastocyst that carried the mosaic or biallelic edit.

### 4.5. Embryo Transfer

Recipient gilts, after estrous cycles were synchronized, were prepared for embryo transfer as described previously [46]. In brief, 0.2 mg of cloprostenol (Planate; MSD Animal Health, Tokyo, Japan) was administered by intramuscular (i.m.) injection to pregnant gilts 35 to 53 days after the day of insemination. Subsequently, a second intramuscular injection of 0.2 mg of cloprostenol and intramuscular injection of 1000 IU of eCG (PMSA for Animal, ZENOAQ, Fukushima, Japan) was administered to the gilts 24 h after the first injection of cloprostenol. At 72 h after the intramuscular injection of eCG, 1500 IU of hCG (Gestron 1500, Kyoritsu Seiyaku, Tokyo, Japan) was administered to the gilts. Approximately 72 h after the hCG i.m. injection, one- to two-cell stage embryos that were electroporated approximately 12 h before embryo transfer were transferred into the oviducts of a recipient gilt under anesthesia. The gilts were placed in the supine position, and the surgical area was disinfected with povidone–iodine (Meiji Seika Pharma, Tokyo, Japan). Approximately 100 zygotes were transferred to each oviduct, resulting in the transfer of 200 zygotes per gilt under sterile conditions.

### 4.6. Mutation Analysis of Piglets Using Deep Sequencing

Ear biopsies were collected from piglets under anesthesia by continuous inhalation of 2–3% isoflurane. Genomic DNA was isolated from the ear biopsies by boiling in a 50 mM NaOH solution. After neutralization, the genomic regions flanking the gRNA target sequences were amplified by two-step PCR using specific primers (Table S2) and the index PCR primers following the manufacturer’s instructions (Illumina, Hayward, CA, USA). After gel purification, the amplicons were subjected to MiSeq sequencing using the MiSeq Reagent Kit v. 2 (250 cycles) (Illumina, San Diego, CA, USA). The mutation rates were defined as the ratio of the number of mutant amplicons to the total read count. A small number of amplicons carrying different sequences that were also detected in WT samples were excluded as sequencing errors. Piglets that carried no WT sequences were classified as having biallelic mutations. Those carrying more than one type of mutation and the WT sequence were classified as mosaics. Piglets that carried only the WT sequence were classified as the WT.

### 4.7. Immunohistochemical Assessment of Piglets

Ear biopsies were collected from pigs, fixed in a 4% paraformaldehyde neutral-buffered solution (Wako, Osaka, Japan), and manually embedded in paraffin. Paraffin-embedded sections were deparaffinized in xylene and rehydrated in decreasing concentrations of ethanol. Blocking treatment was performed by incubation with 10% normal goat serum in phosphate-buffered saline (PBS) for 2 h at 25 °C. To detect the Galα(1,3)Gal epitope, the sections were incubated overnight with 10 µg/mL isolectin B4-Alexa 488 (Thermo Fisher Scientific) at 4 °C. For the detection of Neu5Gc, the sections were incubated overnight with the primary antibody (chicken anti-Neu5Gc polyclonal antibody; clone Poly21469, 146903, 1:100 dilution; BioLegend, San Diego, CA, USA) at 4 °C and were subsequently incubated for 2 h at room temperature with an Alexa Fluor 488-conjugated goat anti-chicken secondary antibody (A-11039, 4 μg/mL; Thermo Fisher Scientific). For the detection of Sd^a^, 20 µg/mL *Dolichos biflorus* agglutinin labeled with rhodamin (RL-1032; Vector Laboratories, Burlingame, VT, USA) was used. Nuclei were counterstained with 4′, 6-diamidino-2-phenylindole (DAPI). The tissues were examined using fluorescence microscopy (BZ-X710; Keyence, Osaka, Japan).

### 4.8. Statistical Analyses

Percentage data for embryos that developed to the blastocyst stage were subjected to arcsine transformation before analysis of variance (ANOVA). The transformed data were evaluated using ANOVA, followed by the protected Fisher’s least significant difference test. The analysis was performed using StatView (Abacus Concepts, Berkeley, CA, USA). The percentage of mutated blastocysts was analyzed using the chi-squared test. Differences with a *p*-value of ≤0.05 were considered statistically significant.

## Data Availability

Data is contained within the article or supplementary material.

## Supplementary Materials

The following are available online at https://www.mdpi.com/1422-0067/22/5/2249/s1.

## Abbreviations

ANOVA	analysis of variance
B4GALNT2	beta-1,4-*N*-acetyl-galactosaminyltransferase 2
Cas9	CRISPR-associated system 9
CMAH	cytidine monophospho-*n*-acetylneuraminic acid hydroxylase
CRISPR	clustered regularly interspaced short palindromic repeat
eCG	equine chorionic gonadotropin
GEEP	gene editing by electroporation of cas9 protein
GGTA1	glycoprotein galactosyl transferase alpha 1,3
hCG	human chorionic gonadotropin
PAM	protospacer adjacent motif
PCR	polymerase chain reaction
TIDE	tracking of indels by decomposition
