# Supplementary material for: One-Step Generation of Multiple Gene-Edited Pigs by Electroporation of the CRISPR/Cas9 System into Zygotes to Reduce Xenoantigen Biosynthesis
Source: Int J Mol Sci. 2021 Feb 24;22(5):2249. doi: 10.3390/ijms22052249 (PMC7956194; doi:10.3390/ijms22052249)
Supplement: Supplementary file 1 [file ijms-22-02249-s001.pdf]

Supplementary Materials:

Table S1. gRNA and Primer Sequences Used for Sequencing Analysis.

Target Gene (Chromosome Localization*)	gRNA					Primer	
	gRNA Name	Target Sequence	PAM	Target	Strand	Forward Primer	Reverse Primer
GGTA1 (Chromosome 1, NC_010443.5)	GGTA1#5	AGACGCTATAGGCAACGAAA	AGG	Exon 2	sense	AAAAGGGGAGCACTGAACCT	ATCCGGACCCTGTTTTAAGG
CMAH (Chromosome 7, NC_010449.5)	CMAH#1	ACATGTTCTTACATGCCTTC	AGG	Exon 1	antisense	GCTGTCAATGCTCAGGGATT	TGCCAAACCTAATTGGGAGA
	CMAH#2	AACATGTGCAAGCACCAAGG	AGG	Exon 1	sense		
	CMAH#3	GAAGCTGCCAATCTCAAGGA	AGG	Exon 1	sense		
B4GALNT2 (Chromosome 12, NC_010454.4)	B4GALNT2#1	TTGAGGATCGACAGACATCT	AGG	Exon 2	antisense	GACCAGACATCGTTCCCAGT	GGGAACTGGCTGTAAAGTGG

*Based on NCBI: Sus scrofa isolate TJ Tabasco breed Duroc, whole genome shotgun sequence, Sscrofa11.1 (GCF_000003025.6)

Table S2. Oligonucleotide sequences used for analysis of the introduced mutations in piglets by deep sequencing

Target Gene	Primer	Common Sequence	Specific Sequence
GGTA1	Forward	ACACTCTTTCCCTACACGACGCTCTTCCGATCT	ATCCGGACCCTGTTTTAAGG
	Reverse	GTGACTGGAGTTCAGACGTGTGCTCTTCCGATCT	CGTGTTCTCTGCCTTGGAAT
CMAH	Forward	ACACTCTTTCCCTACACGACGCTCTTCCGATCT	CCTAATTGGGAGAAAGGATCG
	Reverse	GTGACTGGAGTTCAGACGTGTGCTCTTCCGATCT	AGGGAGGGCTTTCAAACGTA
B4GALNT2	Forward	ACACTCTTTCCCTACACGACGCTCTTCCGATCT	TGCATTTTTGCTCAAGTTGC
	Reverse	GTGACTGGAGTTCAGACGTGTGCTCTTCCGATCT	CACAGTAAAGCCACAGGAGGAG

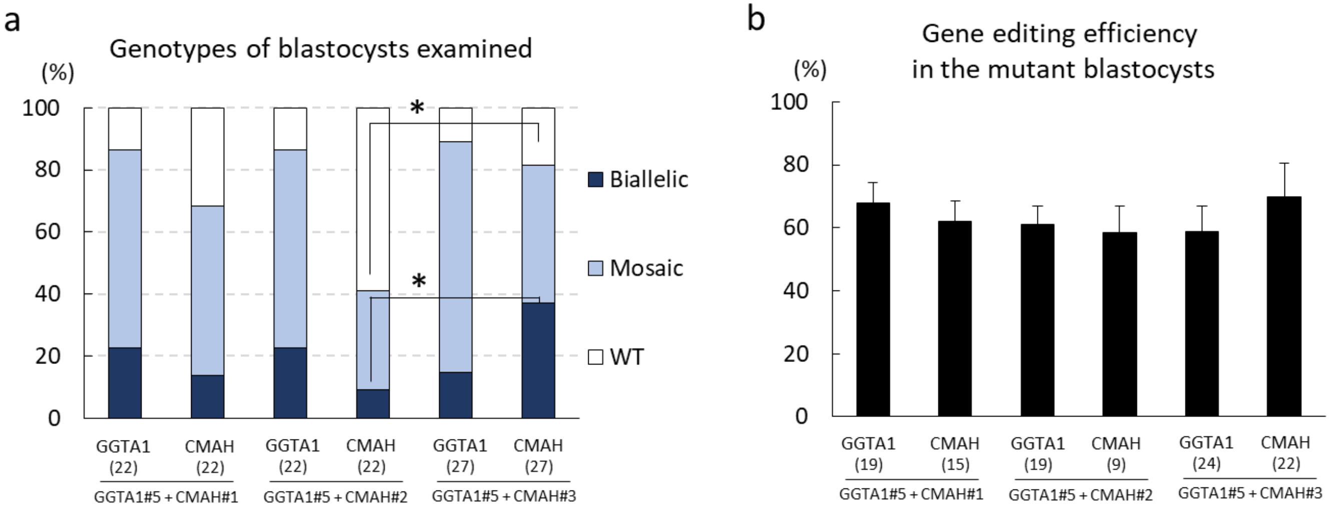

Figure S1. Mutations introduced into each targeting genes by gRNA combinations targeting *GGTA1* and *CMAH*. (**a**) Genotypes of blastocysts were determined using TIDE. Numbers within parentheses indicate the total numbers of examined blastocysts. * $p < 0.05$. WT, wild-type; Biallelic, blastocysts carrying biallelic mutation; Mosaic, blastocysts carrying mosaic mutation. Percentages of blastocysts carrying mutations in *GGTA1* and *CMAH* were analyzed using chi-squared tests. (**b**) Gene editing efficiency in the mutant blastocysts determined using TIDE. Numbers within parentheses indicate the total numbers of examined blastocysts. The editing efficiency was defined as the proportion of indel mutation events in the blastocyst that carried the mosaic or biallelic editing. Means ± SEM are shown.

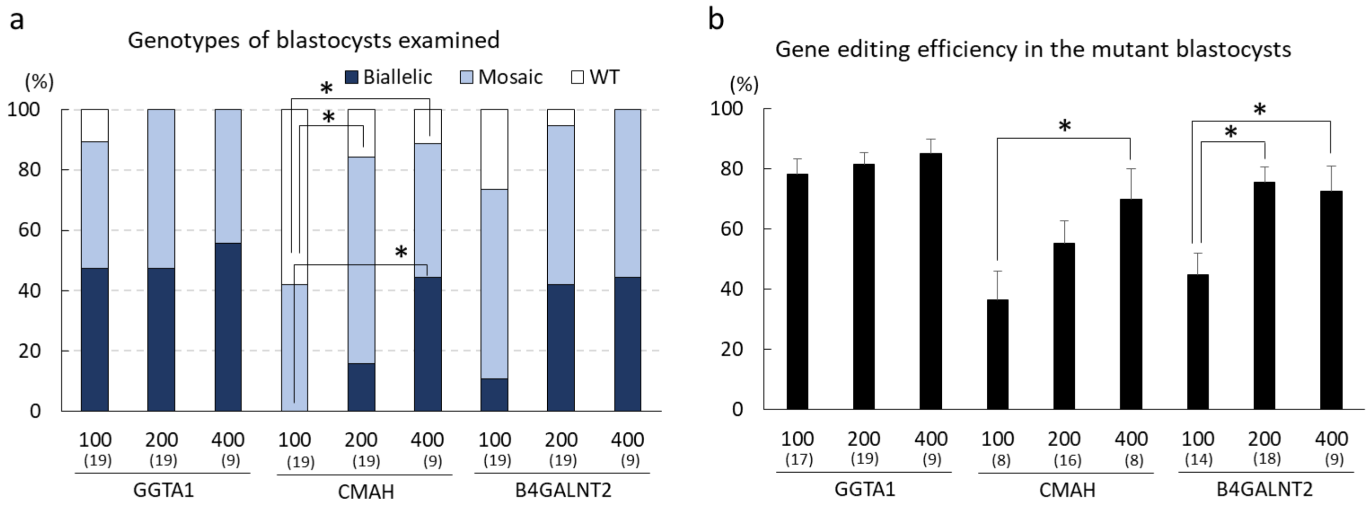

Figure S2. Mutations introduced into each targeting genes by gRNA combinations targeting *GGTA1*, *CMAH*, and *B4GALNT2* with various concentration of Cas9. (**a**) Genotypes of blastocysts were determined using TIDE. Numbers plotted on the horizontal axis indicate Cas9 concentration (ng/μL). Numbers within parentheses indicate the total numbers of examined blastocysts. * $p < 0.05$. WT, wild-type; Biallelic, blastocysts carrying biallelic mutation; Mosaic, blastocysts carrying mosaic mutation. Percentages of blastocysts carrying mutations in *GGTA1*, *CMAH*, and *B4GALNT2* were analyzed using chi-squared tests. (**b**) Gene editing efficiency in the mutant blastocysts determined using TIDE. Numbers plotted on the horizontal axis indicate Cas9 concentration (ng/μL). Numbers within parentheses indicate the total numbers of examined blastocysts. The editing efficiency was defined as the proportion of indel mutation events in the blastocyst that carried the mosaic or biallelic edit. Means ± SEM are shown. * $p < 0.05$.
